# Mucus and Mucins: do they have a role in the inhibition of the human immunodeficiency virus?

**DOI:** 10.1186/s12985-017-0855-9

**Published:** 2017-10-06

**Authors:** Anwar Suleman Mall, Habtom Habte, Yolanda Mthembu, Julia Peacocke, Corena de Beer

**Affiliations:** 1 Division of General Surgery, University of Cape Town and Immune Modulation and Biotherapeutics Discovery, Boehringer- Ingelheim, Danbury, USA; 2 Discipline of Medical Virology, University of Stellenbosch & Tygerberg Hospital, Parow, South Africa; 3 Department of Surgery, Division of General Surgery, University of Cape Town, Observatory Cape, 7925 South Africa

**Keywords:** Mucus, Mucins, HIV-AIDS, Saliva, Breast milk, Cervical

## Abstract

**Background:**

Mucins are large O-linked glycosylated proteins which give mucus their gel-forming properties. There are indications that mucus and mucins in saliva, breast milk and in the cervical plug inhibit the human immunodeficiency virus (HIV-1) in an in vitro assay.

**Main body of abstract:**

Crude mucus gels form continuous layers on the epithelial surfaces of the major internal tracts of the body and protect these epithelial surfaces against aggressive luminal factors such as hydrochloric acid and pepsin proteolysis in the stomach lumen, the movement of hard faecal pellets in the colon at high pressure, the effects of shear against the vaginal epithelium during intercourse and the presence of foreign substances in the respiratory airways. Tumour-associated epitopes on mucins make them suitable as immune-targets on malignant epithelial cells, rendering mucins important as diagnostic and prognostic markers for various diseases, even influencing the design of mucin-based vaccines.

Sub-Saharan Africa has the highest prevalence of HIV-AIDS in the world. The main points of viral transmission are via the vaginal epithelium during sexual intercourse and mother-to-child transmission during breast-feeding. There have been many studies showing that several body fluids have components that prevent the transmission of HIV-1 from infected to non-infected persons through various forms of contact.

Crude saliva and its purified mucins, MUC5B and MUC7, and the purified mucins from breast milk, MUC1 and MUC4 and pregnancy plug cervical mucus (MUC2, MUC5AC, MUC5B and MUC6), inhibit HIV-1 in an in vitro assay. There are conflicting reports of whether crude breast-milk inhibits HIV-1 in an in vitro assay. However studies with a humanised BLT mouse show that breast-milk does inhibit HIV and that breast-feeding is still advisable even amongst HIV-positive women in under-resourced areas, preferably in conjunction with anti-retroviral treatment.

**Conclusion:**

These findings raise questions of how such a naturally occurring biological substance such as mucus, with remarkable protective properties of epithelial surfaces against aggressive luminal factors in delicate locations, could be used as a tool in the fight against HIV-AIDS, which has reached epidemic proportions in sub-Saharan Africa.

## Background

### Introduction

The hydrochloric acid in the mammalian stomach is concentrated enough (pH 1–2) to digest the stomach [1], a finding made by René Antoine Ferchault de Réaumur in the eighteenth century [2]. However the epithelial lining of the stomach remains intact during the digestive process despite the potency of the acid, pepsin and shear associated with digestion [3, 4], behaving, according to Claude Bernard, as if the stomach was made of porcelain [2]!

In 1959 Heatley [5] proposed that mucus on the gastric mucosal surface acted as a mixing barrier and postulated the existence of a pH gradient from the lumen (acid) to the mucosal surface (neutrality). This barrier, later known as the mucus-bicarbonate barrier [3, 6] allowed a restricted mixing of hydrogen and bicarbonate ions [6], in a stable unstirred layer of variable thickness in humans between 50 and 540 μm (mean 180 μm) [7]. The epithelial surfaces of the major internal tracts of the body are protected by a continuous layer of crude mucus gel. The main gel-forming component of the crude mucus gels are mucins (mucous glycoproteins).

Ever since Fultz reported in 1986 [8] that saliva displayed anti-HIV activity, explaining the reason for an absence of transmission of the virus through the exchange of oral fluids [9], it was speculated that a large macromolecular component in saliva, such as salivary mucin, could be responsible for the neutralization of the virus in the oral cavity [10–12]. Further work with crude saliva and its salivary mucins suggested that mucins inhibited HIV-1 in an in vitro assay [13–16]. Anti-HIV activity was also displayed by non-mucin components of saliva such as salivary agglutinin [17] A further investigation of purified mucins from bodily regions of high transmission such as breast milk [18, 19] and the cervico-vaginal area [20] showed that these mucins too were potently anti-HIV-1.

The Human Immunodeficiency virus (HIV) is responsible for a worldwide pandemic, with sub-Saharan Africa carrying the greatest burden of HIV-AIDS. There are approximately 6 million people who are HIV positive over nine provinces in South Africa, with the most number of cases in KwaZulu-Natal, followed by Gauteng province (https://www.commerce.uct.ac.za/Research_Units/CARE/RESEARCH/Papers/Indicators.pdf). Among South Africans aged 15–24 years, 15% of young women and approximately 5% of young men are infected with HIV [21], as compared with 1% of young women and men in the USA [22]. Recent findings show that that women in sub-Saharan Africa substitute penile-anal intercourse (PAI) for vaginal sex for several reasons including as a means of contraception, during menstruation, vaginal infections, money and a belief that it will prevent HIV transmission [23] Understanding risky habits around PAI, such as promiscuity, transactional sex, sex under the influence of alcohol and drugs and the non-use of condom-compatible lubricants, is critical to addressing the spread of HIV and microbicide development [24]. A study in South Africa has shown that the prevalence of HIV infection in women practising PAI was 61.3% compared with 42.7% of women who did not have anal sex, a significant difference [25].

Whilst ongoing unpublished work in our laboratory suggests that pig gastrin mucins (Muc5ac and Muc6) inhibit HIV-1 in an in vitro assay, there is no information in this regard of MUC2/Muc2, the main gel-forming mucin of the lower bowel.

REVIEW (Main Text).

### Mucus

Crude mucus is a complex secretion of primarily water but also proteins, lipids, nucleic acids, cell debris, ions and mucins, to which the stickiness and gel-forming properties of the secretion are attributed [26–29]. It is a visco-elastic secretion, usually continuous over gastrointestinal surfaces [7, 27], with both semi-solid and flow properties, and can reform when sectioned and flow over a relatively long time scale (30 to 120 min) [28–30]. The stickiness of the mucus gel ensures that the gel adheres to mucosal surfaces, providing a slimy coat, for example in the gut, where it facilitates the passage of solid material, whilst protecting the mucosal lining from injury and dehydration [28], and being a platform for the support of a host of antimicrobial molecules [31].

The gastric gel is readily permeable to both H^+^ and HCO_3_^−^ and provides an unstirred layer on the mucosal surface in which the HCO_3_^−^ is secreted by the epithelial cells and is prevented from mixing with the bulk of the acid. Luminal acid is therefore neutralised before it reaches the mucosal cells [3]. Whilst permeable to low molecular weight solutes [32], the gel is impermeable to larger molecules such as pepsin, microbes [33] and certain viruses such as human papilloma virus and Merkel cell polyomavirus which are prevented from reaching the underlying epithelial cells [34]. Pepsin however acts on the luminal surface of the gel and hydrolyses polymeric mucins, liberating degraded mucin subunits into the gastric lumen and causing a complete change in the properties of the gel from an ‘elastic’ (semi-solid) state to ‘viscous’ (liquid) state [35, 36]. Epithelial secretion reinstates the gel to its original thickness, in a state of dynamic equilibrium on the mucosal surface.

Purified mucins have been shown to form a gel in solution [37]. The average concentration of mucin in pig gastric mucus isolated directly from the stomach is approximately 30–40 mg ml^−1^. Molecular interactions between mucins have been shown to occur in this range [37] contributing to a sharp increase in viscosity beyond a glycoprotein concentration of 20–30 mg ml^−1^ and an intrinsic viscosity of 320 ml g-1, suggesting an overlap of individual mucins in solution beginning at 20–25 mg ml^−1^ and resulting in the formation of a gel comprising highly expanded solution-filling mucin macromolecules [27, 37, 38], with an additional contribution through non-covalent interactions [37] by the inter-digitation of oligosaccharide chains of the mucins, involved in the formation of the gel network and contributing to the effectiveness of the gel [27]. Thinner gels of lower viscosity [38, 39], for example in the stomach, reportedly have an increased content of subunit in relation to polymer, and have been shown to be associated with diseases such as gastric ulceration [40, 41], and carcinoma of the stomach [42]. Non-mucin proteins such as trefoil factor (TFF2) have been shown to be physically attached to and strengthen gastric mucus gels, also inhibiting the permeation of H^+^ ions through the gel [43, 44]. It is likely that non-mucin proteins that contribute to epithelial protection such as secretory IgA, lysozyme, gp340 and calcium play a key role in mucus organization [16, 45].

The mucus secretion coats the surfaces of the gastrointestinal, cervical and respiratory tracts [28] as well as the oral cavity [46]. Besides acting as a lubricant on epithelial surfaces, versatility and specialization are features of mucus [47]. The mucus gel barrier on the mucosal surface of the stomach protects it from the shear forces associated with digestion and the potency of hydrochloric acid and pepsin [4, 5]. The muco-ciliary blanket of the respiratory airways protects the alveoli by trapping inhaled dust particles and other foreign impurities which are then removed by swallowing [48]. Colonic epithelium is shielded from hard faecal materials and bacteria by a continuous layer of mucus on the mucosal surface [49, 50]. The mucus plug at the mouth of the cervix protects against the entry of bacteria and facilitates the movement of sperm during the mid-cycle [51]. Saliva aids in the lubrication and homogenization of chewed food [52] and gallbladder mucus protects the underlying epithelium against a concentrated mixture of surface-active chemicals [53].

In each instance, the general but variable properties of mucus are exploited differently to fulfil the special function that is required [47]. Adherent mucus is a ‘true’ gel, that is, it does not dissolve on dilution, even by exposure to denaturants, salts, acid or detergents [54]. Such gels sediment by centrifugation and have cyto-protective properties of an unstirred layer on epithelial surfaces [55].

Mucus layers form an ecological niche for microbiota in the various major internal tracts of the body. Microbes in the mucus gel will be constrained, thus being unable to have interactions such as cell-to-cell communications and inter-species competition [56]. It is also becoming apparent that mucus in the gut is critical to the maintenance of a homeostatic relationship between the gut microbiota, commensal flora dominated by *Fermicutes* and *Bacteriodetes* [57], and their hosts. Deviations from this dynamic interaction have major implications for health, among which are colitis, colorectal cancer and susceptibility to infection [58]. Some gut bacteria have the ability to forage on glycans of the oligosaccharide chains made available by mucins in the gel [59].

### Mucins (mucous glycoproteins)

There are two types of mucins, the secreted [28] and the trans-membrane types [60, 61] types. Evidence suggests that mucin-type O-glycosylation is essential for normal development [62], playing important roles in protein secretion, stability, processing and function, whilst aberrations of O-glycosylation are responsible for certain human diseases and disease susceptibilities [63], with associated modifications in the properties of the mucins [64]. The 4 major secreted gel-forming mucins, which share a common evolutionary ancestor, are MUC5AC in the stomach [65] and airways [66], MUC5B, also in the airways [67] and gall-bladder [68], MUC2 in the colon [69] and MUC6, again in the stomach [70], the genes of which are clustered on chromosome 11p15.5, whilst the gene for the fifth gel-forming mucin, MUC19, is on chromosome 12 [71]. There is a large family of trans-membrane mucins, of which MUC1 and MUC4 play an important biological role in cell-cell and cell-extracellular matrix interactions, in cell signalling and in the biological properties of cancer cells [72]. Currently, the most widely used biomarker for ovarian cancer is MUC16 (CA125), a large transmembrane mucin [73, 74] the levels of which are elevated in 80–90% of patients with advanced ovarian carcinoma. MUC16 lines the endocervix, endometrium, and fallopian tubes to provide an additional barrier to pathogens, preventing them from reaching the epithelium. The endocervix is lined by a single layer of columnar epithelial cells that is highly susceptible to infection by HIV and the MUC16 barrier provides an additional protective layer against infectious agents [75–77]. One study reported that the epidemic disease-causing *S. pneumoniae* species secretes a metalloproteinase, ZmpC, which selectively induces ectodomain shedding of the MUC16, the first line of defence for epithelia in the eye, causing conjunctivitis [78].

MUC16 has been shown to have an enhanced binding of IgG antibodies generated during chronic HIV infection [76]. This interaction between antibodies and MUC16 has potential to concentrate HIV-specific antibodies within the glycocalyx covering the luminal surface of the endocervix, thus enhancing mucosal barrier function and having implications for the design of the next generation vaccines and microbicides [76].

Changes in the sequence of glycosylation of mucins in different settings generate a variety of epitopes in the oligosaccharide side-chains of mucins, including newly expressed blood-group antigens, distinguishing between normal and diseased states. Tumour-associated epitopes on mucins and their antigenicity make them suitable as immuno-targets on malignant epithelial cells and their secretions, creating a surge of interest in mucins as diagnostic and prognostic markers for various diseases [79], and even influencing the design of mucin-based vaccines [80].

It is known that oncogenic changes are associated with changes in glycosylation in glycoproteins and glycolipids [81], generating new antigens exploitable as laboratory diagnostic markers, amongst which are T, Tn and sialyl-Tn antigens, in a variety of cancers [82]. The sialyl-Tn epitope is one of the most specific tumour antigens described so far, being highly expressed on many adenocarcinomas but having a very limited expression in normal adult tissues [83]. Its expression on mucins in the non-dysplastic colonic mucosa of long-standing ulcerative colitis patients identifies an increased cancer risk [84]. A variety of epitopes are expressed on the mucin oligosaccharide chains, glycan motifs that can be recognised by bacterial adhesins and lectins [85] and a host of mucin-microbe interactions have been reported [86]. Adhesins allow for interactions between viral haemagglutinins and terminal sialic acid residues in mucins. Salivary and respiratory mucins in cystic fibrosis have modifications of their carbohydrate side-chains that increase their binding affinity for Pseudomonas aeruginosa [87]. It is likely that the primary function of the oligosaccharide diversity in mucins, for example in the respiratory airways, is to enhance the possibility that bacteria bind to mucus, thus facilitating their removal by mucociliary transport. Mucins provide competing receptors for cell-surface glycoconjugates, in this way trapping bacteria and preventing them from colonizing the epithelia [31, 88].

Many monoclonal antibodies reactive with cancers detect mucin carbohydrate determinants which on malignant cells are associated with cancer aggressiveness and metastasis. Some of these epitopes are cryptic but can be exploited by directing immunotherapy against them, since they are potentially immunogenic and can stimulate humoral and cellular immune responses in cancers and perhaps HIV-AIDS? One such antigen is the Thomsen-Friedenreich (TF) antigen, found on mucins and which was reported to protect against a lethal TA3-Ha tumour transplant in mice [89].

In the human stomach alone more than 70 different oligosaccharides are carried by mucins, mostly neutral and highly fucosylated, with less sialyated and no sulphated groups, suggesting an enormous diversity in a single organ [90]. *Helicobacter pylori* is a known causative agent for chronic active gastritis, ulceration and carcinoma of the stomach [91]. It is found within the mucus gel, where its attachment is mediated by the Lewis^b^ antigen in MUC5AC, and it is also attached to mucosal epithelia. Sialic acids, found both in mucins and on the epithelial surface, seem to be the favoured binding site of various viruses such as influenza viruses, reoviruses and adenoviruses [92]. It is apparent that mucin glycans, their length, composition and sequences are subject to alterations in different environmental conditions [93], during which they may be either protective by binding and retarding the movement of these organisms through the gel towards the epithelia [34], or they may succumb to infection.

### Components of saliva

An amount of approximately 1500 ml of saliva [94], a dilute aqueous secretion which contains 99% water, mucins, lipids, and proteins [46, 95, 96] is produced daily by the submaxillary, sublingual and parotid glands [97]. Saliva lubricates and cleanses the oral cavity [98], provides an innate immune defense against pathogens [99] and plays an important role in the aggregation and clearance of micro-organisms, including HIV [100, 101]. Most of the anti-bacterial proteins in saliva also display anti-viral activity [102]. Oral transmission of HIV by exchange of oral fluids is a rare event, even in cases of bleeding and the presence of exudate in the oral cavity. Only 1–5% of an HIV - positive patient’s saliva contains infectious HIV despite there being higher levels in the blood. It is thought that hypotonic disruption may be a major mechanism by which saliva kills infected mononuclear leukocytes and prevents their attachment to mucosal epithelial cells and production of infectious HIV, thus preventing transmission [9]. Any remaining infectious HIV will have to be inhibited by salivary components, the search for which began in the 1980s.

The recent concept of salivaomics, a broad collection of technologies investigating salivary constituents and their roles in saliva, encompasses genomics, transcriptomics, proteomics, metabonomics and microbiota studies, with saliva having applications in early diagnosis of diseases, and in translational and precision medicine [97, 98]. A consortium of three research groups identified 1116 proteins in saliva, a high proportion of which were found in plasma and tears [103] making saliva an excellent substitute for blood as a diagnostic fluid, easy to collect repeatedly, non-invasively, and with the opportunity of multiple sampling by patients themselves, thus reducing clinical costs and increasing patient compliance [104].

### Salivary mucins

Human saliva was originally reported to contain two carbohydrate rich mucin populations, namely MG1 encoded by the MUC5B gene and MG2, encoded by the MUC7 gene [105]. These mucins, synthesized by different cells of the salivary glands [94], are reported to differ structurally and functionally [67, 68] and are separable by Sepharose 4B gel filtration [13, 95, 105].

MUC5B [105–107] is one of the most widely distributed mucins found in saliva and the respiratory and female reproductive tracts. Secreted by all the salivary glands except the parotid, MUC5B is known to contain 19% protein and 81% carbohydrate [107, 108]. Salivary MUC5B is a large, polymeric disulphide bonded secreted mucin with a complex structure [83], the carbohydrate moiety of which is reported to aggregate and clear bacterial, fungal and toxic pathogens from the oral cavity [96, 103, 105], and to inactivate HIV [13]. It is conceivable that large disulphide-bonded mucin polymers which form non-covalent interactions through the inter-digitation of their densely packed oligosaccharide side-chains [27] result in the formation of a network that surrounds microbes thus restricting their movements. It is not known how these constraints influence microbial interactions, such as cell-to-cell interactions and competition among the vast number of organisms that live in mucus [56]. In the case of microbiota in the oral cavity, salivary MUC5B affects intra-species interaction by promoting dispersal of bacteria and fungi. In an experimental, dual-species model Frenkel and Ribbeck [56] very recently showed that mucins, in this case salivary MUC5B, promoted the coexistence of two competing bacteria, *S. sanguinis* and *S. mutans*, perhaps by promoting a less competitive mode of growth between them.

MUC7 [106] is found in saliva [107] and together with MUC5B in the bronchus [109]. Salivary MUC7, the smallest of the secreted mucins, is a non-gel forming, less heterogeneously glycosylated mucin [110] secreted by the submandibular, sublingual and palatine salivary glands [111]. MUC7 is reported to contain 30% protein and 68% carbohydrate [108, 111]. Thus far two glycoforms of MUC7 namely MUC7a and MUC7b with similar amino acid but different sialic acid and fucose compositions are reported to exist in human saliva [107, 112]. MUC7 is reported to possess inhibitory activity against a number of bacterial, fungal and viral strains including *Streptococci, P.aeruginosa, S. aureus, C. albicans and S. cerevisiae*, amongst others [13, 112–114]. It also plays a crucial role in the inhibition of HIV transmission through saliva [10, 13].

A gene for a putative polymeric mucin, MUC19, reported as a fifth human gel-forming mucin, has been shown to be expressed in human salivary glands [71]. Rousseau et al. [52] showed that there was no evidence for MUC19 in human saliva, a finding confirmed in our laboratory (Santhoshan Pillay, personal communication), suggesting that MUC19 is not a major component in human saliva.

### Anti-HIV activity of human saliva

The idea that incubation of HIV with saliva decreases HIV activity was first suggested by Fultz in 1986 [8], who showed that whole saliva and saliva filtrates inhibit HIV. For complete inhibition to occur, virus and saliva had to be incubated together for more than 30 min. This finding led to more investigations by various groups to identify the factor/s in saliva responsible for the inhibition of HIV. Whole saliva and equal volumes of HIV-1, when filtered through 0.45 μm Nalgene filters, showed no toxicity to phytohemagglutinin – activated normal peripheral blood lymphocytes and showed inhibition of infection of these lymphocytes [10]. They further showed that the inhibition was also by submandibular secretions and not those of the parotid gland, a finding confirmed by Archibald and Cole [11]. Filtration studies using whole saliva and an HIV-1/ATH8-cell cytopathic system and the monitoring of inhibitory activity by viable cell counts and HIV-1 core antigen and reverse transcriptase levels, showed approximately 50% less virus than in HIV-1/media filtered controls, suggesting that inhibition was caused by aggregation of the virus by an unknown salivary component [115]. Filtration of whole saliva and human submaxillary and sublingual (HSMSL) saliva through a 0.22 μm pore size filter, prior to addition of the virus showed far less inhibition of HIV-1 in the filtrate as compared to the virus being incubated with saliva prior to filtration [116]. It was Malamud et al. in 1993 [100] who reported that the major anti-HIV activity in saliva resided in the submandibular secretion in which inhibition is seen in 2 min and increased with time. This inhibitory activity was specific for HIV and shows no effect on HSV and adenovirus. Electron microscopic studies showed that HIV-saliva aggregates were trapped in 0.45 μm pore size nitrocellulose filters suggesting that human submandibular saliva aggregates HIV [100, 117]. Robinovitch et al. [118] submitted saliva in blinded fashion for quantitation of their anti-HIV activity, using a syncytia-forming MT-2 cell assay or the p24 antigen ELISA. Nine out of the 27 subjects showed detectable anti-HIV infectivity activity. Parotid secretions in this study showed no inhibition and filtration of the saliva through an Amicon 10 filter before incubation with the virus once again abolished inhibitory activity. Similar studies using two other biological fluids, urine and cerebrospinal fluid, revealed no anti-HIV infectivity activity. These findings confirm the presence in saliva of inhibitory activity directed toward HIV by an unknown component, again in whole saliva and by inference, submandibular secretions [118].

The suggestion that mucins could be the anti-HIV factor was first made by Bergey et al. [119], who fractionated human submaxillary/sublingual saliva (HSMSL) and demonstrated anti-HIV activity in mucin-rich fractions. Electron micrographs revealed an association of viral particles with salivary sediment, again suggesting an aggregation of virus by salivary components, whilst gel filtration studies showed that inhibition was associated with mucin-rich fractions of saliva. Shine et al. [12] reported that viral p24 and HIV-1 RNA were detected in tonsils and adenoids of asymptomatic seropositive individuals and yet the infectivity of whole saliva, that from the HSMSL, and less so the parotid glands, reduced HIV-1 infectivity in vitro. Malamud et al. [120] suggest that multi anti-viral activities are present in saliva, working separately or together to reduce HIV infectivity, the effect of which is directly on the virus rather than the infected host cell. Salivary agglutinin [120, 121], secretory leukocyte protease inhibitors (SLPI) [122] and proline-rich proteins [118, 123], are examples of such anti-HIV factors in saliva [11]. The study of Baron et al. [9] strengthened this multi anti-viral idea by adding the hypotonic nature of saliva which causes the disruption of infected leukocytes, perhaps producing free virus into the oral cavity to be acted upon by other protective factors such as salivary mucins, further reducing any chance of transmission through exchange of oral fluids. Nagashunmugam et al. [14] reported that though the level of anti-HIV-1 activity was higher in submandibular than parotid or whole saliva with or without filtration (in 9/15 cases), there was some inter-individual variation in that 4/15 showed lesser inhibitory activity, and 2/15 none at all except after filtration. They went further to show that saliva rendered HIV-1 harmless by stripping gp-120 from the virus, with partial purification by anion – exchange chromatography revealing two high - molecular – weight sialyated glycoproteins, salivary agglutinin and mucin as the possible factors of inhibition of HIV [15]. Further studies confirmed that whole saliva and individual secretions of submandibular and sublingual but not those of the parotid glands (which lack mucin) [94, 124, 125], are inhibitory, suggesting that the virus is rendered harmless when bound to a high-molecular weight salivary component, the complex being separable by filtration [11, 12, 14, 15, 100, 122, 125]. Bergey et al. in 1994 [119] demonstrated a maximum anti-HIV-1 activity of the mucin - rich fractions of human submandibular saliva. This reduces the risk of the transmission of HIV through the oral route, raising questions about the specificity of inhibition in the oral cavity [9, 12, 100, 101, 119]. The detection of HIV infection with an oral fluid-based (saliva) rapid and accurate point-of-care test is already a reality [126].

Work in our laboratory confirmed the anti-HIV-1 activity of crude human saliva and its purified mucins MUC5B and MUC7 from uninfected individuals [13, 16, 127] by in vitro assays, according to the method of Nagashunmugam et al. [14]. The virus used in the assay was first isolated and fully characterized and sequenced in the Department of Medical Virology, University of Stellenbosch in February 1986 (CdB) [13, 128]. Habte et al. [128] further showed that MUC5B and MUC7, purified from the saliva of infected individuals of different CD4 counts (< 200, 200–400 and >400) did not inhibit the virus in an in vitro assay. Saliva and purified salivary mucins were incubated with HIV-1 prior to infection of the human T lymphoblastoid cell line (CEM SS cells), after which viral replication was measured by a p24 antigen assay. This finding was subsequently refuted by Peacocke et al. [16] in a wider study, and one which was a slight variation of the Nagashunmugam et al. [15] assay, comparing samples from HIV positive and negative individuals, incubated with subtype C HIV-1 and infection of peripheral blood mononuclear cells (PBMCs) [16]. Peacocke et al. [16] reported that crude saliva and its salivary mucins from both uninfected and HIV positive individuals inhibited, HIV-1 in an in vitro assay.

### Breast milk components

Human breast milk is the culmination of 200 million years of Darwinian pressure on mammalian lactation as the sole source of early infant nourishment [129]. Human milk is an excellent example of a mutual and beneficial relationship of humans and microorganisms, contributing to both acute survival and long-term health of humans and ensuring the selective colonization and support of a protective microbiota [129]. The most abundant component of human milk are lactose, lipids and 200 molecular species of oligosaccharides, the latter being indispensable for the nourishment of the infant’s microbiota, the general growth of the infant, the stimulation and modulation of its immune system, cognitive development and protection from toxins and disease. The oligosaccharide population is remarkably diverse and displays an inter-individual variation during lactation amongst feeding mothers [129]. Human breast milk is also reported to contain a number of non-immunological components such as glycolipids, glycoproteins, mucins and glycosaminoglycans [130–133] and proteins which include lysozyme and β-casein [131]. These components are biologically active and protect breast fed infants against harmful microbes, viruses, and toxins [130, 132], including the inhibition of rotavirus and S-fimbriated *E. coli* by milk mucin, inhibition of cholera and labile toxins of *E. coli* by milk glycolipids [133], inhibition of *S. pneumoniae*, *H. influenzae* and entero-pathogenic *E. coli* adherence by human milk oligosaccharides [134] and quite recently, neutralization of HIV-1 by tenascin-C [135]. Breast milk components are also reported to inhibit pathogens such as hepatitis C, Ebola, cytomegalovirus, Dengue virus, *M. leishmania, C. albicans,* and *H. pylori* [131, 132].

### Human breast milk mucin

Human milk contains well characterised MUC1 [136] and MUC4 [137], both trans-membrane mucins [138], which are components of the milk fat globule membrane [130, 139]. MUC1, which is located on chromosome 1q21-q24 [140], is the most widely distributed human mucin and is expressed in the mammary gland, lungs, pancreas, gastrointestinal tract, kidney, bladder, endometrium, ovary and testes [141]. It has 25 to 125 tandem repeats and has a molecular weight between 250 and 1000 kDa [141, 142], with carbohydrate comprising 50% of its weight [143].

MUC4 is expressed in the ciliated and goblet cells of the epithelial tissues of the stomach [144], breast, endocervix, colon [145], uterus, ovary, salivary gland, prostate, thyroid, mammary gland, esophagus, testis and placenta [106] and tears [146]. Beyond the respiratory tract, MUC4 has been reported to be a novel prognostic factor in patients with invasive ductal carcinoma of the pancreas and chronic pancreatitis [147, 148], intra-hepatic cholangiocarcinoma-mass forming type [149], extra-hepatic bile duct [150] and lung adenocarcinoma [151] and, unlike MUC1, is not associated with metastasis in cholangiocarcinoma [152].

### Anti-HIV activity of breast milk and its mucins

There have been contradictory findings with respect to the capacity of breast milk to inhibit HIV-1. A study by Wahl et al. [153] suggested that prior to their work, breast milk studies in relation to HIV inhibition were in vitro studies which all showed milk to have a strong inhibitory effect on HIV infectivity. However Habte et al. [18] showed that crude uninfected breast milk, did not inhibit HIV-1 in an in vitro assay, a finding confirmed by Mthembu et al. [19] for both HIV negative and HIV positive milk, in our laboratory. Purified MUC1 isolated from the breast milk, inhibited HIV-1 [18]. Another study by Newburg et al. in 1992 [154], found breast milk from both HIV positive and negative patients to contain an HIV-inhibitory factor, not present in bovine milk or human sera, that inhibited the binding of HIV epitope-specific MAb to recombinant CD4 receptor molecule, and which lost its potency upon destruction of sulphate molecules, suggesting it could be a mucin.

On the other hand Kazmi et al. (2006) [155] demonstrated high levels of anti-HIV-1 activity by crude breast milk comparable to that of saliva. It was also found that breast milk obtained from uninfected women had a primary HIV-1 neutralizing protein, tenascin-C, which captures and neutralises HIV-1 by binding to the chemokine co-receptor binding site on the HIV-1 envelope, is an extracellular matrix protein important in foetal development and wound healing [135]. The complexity of this issue is highlighted by the finding of Lyimo et al. [156] that breast milk may provide a protective function against cell-free HIV-1 but may be less effective at blocking infection by cell-associated virus. When human milk was inoculated with cell-free and cell-associated HIV-1 and subsequently heated and pasteurised, both forms of the virus were inactivated [157].

Furthermore, in the absence of anti-retroviral prophylaxis, greater than 90% of infants exposed to HIV-1 breast-feeding remain uninfected, despite daily mucosal exposure to the virus for up to 2 years [135]. It is known that despite the nutritional and health benefits of breast milk and its protective role against a variety of pathogens [158], breast milk can serve as a vector for mother-to child HIV transmission [159], with reports that breast feeding was responsible for about 30–50% of approximately 500,000 infant HIV-1 infections worldwide around the turn of the century [158, 159], or generally at rates of 20–50% [154].

Although HIV-1 is inhibited by saliva [13, 16] it is shown that the saliva of the infant is overwhelmed by the viral load in the milk of the mother during breast feeding [160]. Human breast milk has a high titre of viable virus ranging from 240 to 8100 copies/ml, compared with less than 1 copy/ml in saliva [156]. However there is still a case for breast-feeding as opposed to replacement feeding by using antiretroviral drugs and shortened breastfeeding, which markedly decrease breastfeeding HIV-1 transmission, shifting the balance to make replacement feeding less beneficial [159, 160].

The paradoxes in the findings mentioned above could complicate the well-established notion of ‘breast-is-best’, especially in the context of an HIV pandemic in resource limited settings such as in sub-Saharan Africa. It is known that most HIV-infected infants acquire HIV through breastfeeding, over and above infection in utero and during labour and birth. However, most breastfed infants remain uninfected despite prolonged and repeated exposure to HIV. All this suggests that breast milk in relation to HIV has contradictory roles, providing protection or is a risk for infection [153].

Wahl et al. [153] saw the necessity of examining the inhibitory effect of breast milk in an in vivo humanized (BLT) mouse model of oral HIV transmission. They showed that breast milk has a strong inhibitory effect on oral transmission of cell-free and cell-associated HIV. Furthermore anti-retrovirals administered prior to oral transmission of HIV prevented transmission in BLT mice.

The WHO in 2014 reported that an estimated 240,000 children become infected with HIV annually through breastfeeding but still advised that in resource-limited settings (which sub-Saharan Africa and Southern Africa are), mothers should still breastfeed, in combination with infant or maternal antiretroviral therapy, because the health benefits of breastfeeding far outweigh its risks [160]. Most infants breastfed by HIV positive women do not become infected, even when fed HIV positive milk [160]. They further showed that the amazing anti-HIV properties of breast milk inhibit other modes of infection, preventing multiple routes of infection, for example preventing vaginal transmission [160].

Both Habte et al. [18] and Mthembu et al. [19] speculated that crude breast milk did not inhibit HIV-1 in vitro because of the membrane-bound nature of breast milk mucins, which are enclosed in fat globules, thus preventing contact between the virus and the mucin. Purified MUC1, when isolated from crude normal breast milk after delipidation inhibited HIV-1 [18] and showed anti-poxvirus activity [138], both in an in vitro assay. It has also been shown that MUC1 prevented DC-SIGN-mediated transmission of HIV-1 from dendritic cells to CD4+ T cells [160]. A follow-up and broader study in our laboratory by Mthembu et al. [19] comparing the inhibitory potential of normal milk and milk infected with HIV-1, showed that crude human breast milk from both HIV positive and HIV negative patients did not inhibit HIV-1, whilst its purified mucin components (MUC1 and MUC4), from normal and infected milk, did. More studies would be required to clarify the differences in these findings.

### Cervical mucus

Cervical mucus is known to play a crucial role in human reproductive physiology by providing lubrication for and the prevention of the desiccation of the vaginal mucosa [161–164] preventing microbial colonization, fluid loss and the control of sperm survival and migration to the upper reproductive tract [163, 165–167]. According to Yurewicz and Moghissi [168], cervical mucus facilitates the movement of sperm to the upper reproductive tract during the mid-cycle and, under the influence of oestrogen, selectively excludes abnormal and poorly motile sperm cells. Mucus enhances the survival of sperm by protecting it from phagocytosis [169] and the anti-HIV activity of mucus is at its best at an acidic pH [170]. Despite the continuous change in mucus viscosity throughout the menstrual cycle and the risk during the proliferative phase of pathogen entry into the cervix, the mucus clears millions of micro-organisms daily from the female reproductive tract [171]. The cervical mucus plug is a viscous gel both on the mouth of the cervix and in the cervical canal, and which is released during labour [20]. One study reported that the plug inhibits but does not block the passage of *Ureaplasma parvum* when it ascends from the vagina to the cervix [172]. It is thought that the mucus barrier provides protection to the female reproductive tract through a stable association between IgG and IgA with cervical mucus and an association of IgG only with cervicovaginal mucus [164]. Vaginal microbiome dysbiosis is associated with HIV infection and alterations in the levels of gel-forming mucins in the vagina have been reported, with a significant increase of MUC5B levels in a *Lactobacillus crispatus* environment and a relative increase in MUC5AC in moderate and severe dysbiosis, dominated by *Lactobacillus iners* [173].

There is a varying amount of mucus during the human reproductive cycle, which is differentially abundant during the oestrus (Day 0 of the cycle) and luteal phases (Day 10), contributing to the varying viscosity of the cervicovaginal fluid. The first physiological barrier the spermatozoa encounters is the cervix with its vaginal side covered by a highly viscous mucus, the cervical vaginal fluid (CVF) [174]. The amount of CVF increases at the time of ovulation concomitantly with a higher state of hydration and a reduced viscosity, to facilitate sperm migration [175]. The mechanical properties of the mucus are essential to select spermatozoa with the highest fertilizing ability, i.e. with normal morphology and efficient mobility [176]. Thus, only a small percentage of normal spermatozoa will be able to cross the cervical mucus and fill the lumen of the cervix. The rheological properties of this mucus are known to change along the cycle, with a reduced viscosity at oestrus, to allow spermatozoa to reach the uterus, and an increased viscosity during the luteal phase to protect the uterus from external contamination [175]. The viscosity of the cervical mucus is mainly due its high content of mucins, 5B and 5AC [177]. Gipson et al. [178] using the MUC5B antibody along with a cervical mucin standard cervical mucin isolate in enzyme-linked immunosorbent assay, showed that a peak of MUC5B mucin in human cervical mucus collected at midcycle, compared with mucus from early or late in the cycle. This peak in MUC5B content coincides with the change in mucus character that occurs at mid-cycle, suggesting that this large mucin species may be important to sperm transit to the uterus [178].

### Anti-HIV activity of purified cervical mucins

Lai et al. [170] showed that CVF, obtained from donors with normal lactobillus-dominated vaginal flora, efficiently traps HIV, causing it to diffuse more than 1000 fold more slowly than it does in water. This is due to the acidification of the mucus by *Lactobacilli*, which produce lactic acid which acts by abolishing the negative surface charge on HIV without lysing the virus membrane, and facilitating its entrapment in the mucus [170]. In a study using human cervical explants and in vivo exposure to HIV-1 in a rhesus macaque vaginal transmission model, it was shown that HIV-1 diffuses through the squamous epithelium, penetrating areas where cell junctions were absent but unable to traverse a mucus barrier to enter columnar epithelial cells [179]. Habte et al. [20] in our laboratory showed that the crude cervical plug mucus obtained from uninfected mothers prior to delivery, did not inhibit the virus in an in vitro assay but the purified mucin from the same plug, did [20, 127]. A study by Ghosh et al. (2010) [180] showed that CVL secretions from HIV+ and HIV- women contain innate and adaptive factors inhibiting the virus. Further work in our laboratory showed that crude cervical mucus from HIV positive and negative individuals did not inhibit the virus but their purified mucins did (manuscript in preparation).

Chen et al. [181] hypothesised that reducing HIV heterosexual transmission at the portal of entry would be the best prevention strategy. Since the affinity of antibodies for mucus is weak, they developed an in vitro model in which multiple antibodies, binding to a single virion at one time would increase the overall antibody-mucin binding avidity, creating an inheritable virus-mucin affinity. The model predicts that HIV-specific Ab in cervico-vaginal mucus will lead to the formation of an HIV concentration front near the semen/CVM interface, away from the vaginal epithelium, thus minimizing the risk of infection. This model they say is a first step toward an improved quantitative understanding of the dynamics of mucosal immunity in the female reproductive tract and future improvements to the model model will provide further predictive insights into reinforcing vaginal mucosal immunity against HIV and other sexually transmitted infections.

### Possible applications of the role of mucus and mucins against HIV-1

Work in our laboratory on the role of mucus and mucins in HIV-AIDS suggest that crude saliva and purified mucins from saliva [13, 16, 128], breast-milk [18, 19] and the cervical plug [20] inhibit the HIV virus in an in vitro assay and that it is the inhibitory properties of these mucins in crude saliva that prevent the transmission of the virus during the exchange of oral fluids. The visco-elastic and gel-forming properties of crude mucus gels, which provide protection of epithelial surfaces against hostile milieu in the major tracts of the body, are due to their mucin content [35, 37] or an interaction between mucins and non-mucin protein [45]. Furthermore mucins by themselves can form gels in vitro at concentrations comparable to those in the physiological context [35].

Lieleg et al. [34], in a study of porcine gastric mucins, suggested that these mucins might be suitable candidates for supplements in personal hygiene products such as mouth rinse, toothpaste and perhaps even a genital paste, because of their anti-viral activity.

Efforts to elicit HIV glycan-dependent broadly cross-neutralizing antibodies by vaccination have not been successful [34]. Microbicides have been in development for over two decades and early efforts in this regard failed due to irritation of the vaginal epithelium and subsequent inflammatory responses [182, 183]. Karim et al. [184, 185] developed an antiretroviral microbicide, Tenofovir gel providing proof of concept that such formulations are possible in the fight against the AIDS pandemic and the empowerment of women whose vulnerability to infection in this situation is obvious. The gel has been reported to reduce HIV acquisition by an estimated 39% overall, and by 54% in women with high gel adherence [184].

In the light of the sheer importance of continuing to meet this challenge and the above-mentioned protective properties of mucins together with their additional anti-HIV-1 effects, we wondered whether mucins could be exploited to formulate a biologically based biocompatible substance that could act as a topical prophylactic to prevent the transmission of the virus during vaginal and anal sexual intercourse (see ref. [34]). The increase in heterosexual anal intercourse reported in sub-Saharan Africa [23–25] with the increased possibility of transmission of HIV via the anal route, has given the question of microbicide development a greater urgency [186]. MUC2 is the gel-forming mucus in the large bowel and its anti-HIV properties have not as yet been studied and we plan to embark on such a project in the near future. Further work is required to elucidate the mechanism of the interaction between mucin and HIV-1. Our studies and those of others suggest a strong possibility that any interaction between mucins from different areas in the body and HIV-1, would take place at the oligosaccharide level. There is a huge absence of carbohydrate research facilities in South Africa generally and we are planning collaborative efforts with groups overseas. Our focus would be on the sequences of the mucin sugar side-chain that gives optimal binding and inhibition of HIV-1. Thus far inhibition by salivary MUC5B has shown great promise in this regard. Factors such as the diurnal variation of mucus-mucin secretions and the health and nutritional status of the donor will be taken into account during sample collection. We are currently working towards comparing the anti-HIV potency of each of the gel-forming mucins, individually and in various combinations, which will provide the basis of our planned formulation of a vaginal paste that will further fortify the vaginal epithelium against infection during sexual intercourse. The increase in penile-anal sexual intercourse among African women raises questions about the role of the mucus gel covering the gastrointestinal mucosa in this context. MUC2 is the gel-forming mucin in the lower bowel [24, 36] and our aim to test individual mucins against HIV-1 will be informative in this regard. Because of the difficulties of accessing human material for our work, we have embarked on another project to test the anti-HIV potency of mucus material obtained from animal sources.

## Conclusions

Whilst ongoing unpublished work in our laboratory suggests that pig gastrin mucins (Muc5ac and Muc6) inhibit HIV-1 in an in vitro assay, there is no information in this regard of MUC2/Muc2, the main gel-forming mucin of the lower bowel. Considering the high prevalence of HIV-AIDS in sub-Saharan Africa and the changing sexual practices such as PAI amongst certain groups, with their associated risk of more infections, we wondered whether the gel-forming and protective properties of mucus, together with suggestions of the anti-HIV activity of mucins, could be exploited in the formulation of a mucus-based microbicide? This would help serve to fortify existing mucus gel linings in the vagina and anus together with providing a scaffolding for smaller molecules with anti-HIV properties, to reduce the risk of infection.
